# Tissue-Specific VDR Pathway Gene Expression Is Not Associated with Circulating 25OHD in Adolescents with Severe Obesity

**DOI:** 10.3390/metabo16090627

**Published:** 2026-08-29

**Authors:** Olivia Z. B. Ginnard, Maria Morales, Gabrielle Phillips, Mary L. Brandt, Sridevi Devaraj, Alexis Wood, Stephanie R. Sisley

**Affiliations:** 1 USDA/ARS Children’s Nutrition Research Center, Baylor College of Medicine, Houston, TX 77030, USA; maria.morales@bcm.edu (M.M.); gphillips193@aol.com (G.P.); alexis.wood@bcm.edu (A.W.); stephanie.sisley@bcm.edu (S.R.S.); 2 Texas Children’s Hospital, Baylor College of Medicine, Houston, TX 77030, USA; devaraj@bcm.edu; 3 Department of Surgery and Center for Medical Ethics and Health Policy, Baylor College of Medicine, Houston, TX 77030, USA; mary.l.brandt@gmail.com

**Keywords:** vitamin D, obesity, gene expression, bariatric surgery

## Abstract

**Highlights:**

**What are the main findings?**

- VDR-target genes demonstrated correlated expression patterns within metabolically relevant tissues in individuals with obesity, despite being involved in distinct biological pathways.
- Circulating serum 25OHD levels were not significantly associated with tissue-specific VDR pathway gene expression in individuals with obesity.

**What are the implications of the main findings?**

- The absence of a statistically significant association between serum 25OHD and tissue-specific VDR-target gene expression suggests that circulating vitamin D status may not fully reflect tissue-level vitamin D biology in severe obesity.
- These findings support further investigations of tissue-specific VDR pathway gene expression responses to vitamin D supplementation in obesity.

**Abstract:**

**Background/Objectives**: Vitamin D deficiency is highly prevalent among children with obesity, but the mechanisms underlying the effectiveness of vitamin D supplementation remain poorly understood. This study examined the expression patterns of VDR-target genes across metabolically diverse tissues and compared these molecular measures with circulating serum 25-hydroxyvitamin D (25OHD), the current clinical marker of vitamin D status. We hypothesized that VDR-pathway gene expression would correlate within metabolically relevant tissues but would not be significantly associated with circulating serum 25OHD levels. **Methods**: A secondary analysis was performed on blood, intestinal, and visceral and subcutaneous adipose tissue (VAT and SAT, respectively) samples obtained from adolescents with obesity. Subject data included age, gender, race/ethnicity, and BMI. The tissues were analyzed via real-time qPCR to obtain quantitative levels of VDR-target gene expression, which included *TLR4*, *THBD*, and *VDR* in SAT and VAT and *TRPV6*, *S100G*, and *VDR* in intestinal tissue. Blood samples were analyzed for serum 25OHD. **Results**: Gene expression of *THBD*, *VDR*, and *TLR4* in SAT and VAT significantly correlated with each other. In intestinal tissue, there was significant correlation between *TRPV6*, *S100G*, and *VDR*. No statistically significant associations were identified between the gene expression levels and serum 25OHD levels. **Conclusions**: VDR-target gene expression levels correlated with each other across diverse tissues but not with serum 25OHD levels. This discrepancy suggests that circulating 25OHD concentrations may not fully reflect vitamin D action.

## 1. Introduction

Vitamin D regulates a wide range of physiological processes in children that are vital to human health. Vitamin D’s actions extend beyond its classic role in calcium homeostasis and bone health, including blood pressure, glycemic control, immunity, and metabolic health [1,2,3,4,5,6,7,8,9]. Following its conversion to its active form of 1,25-dihydroxyvitamin D (1,25OHD), vitamin D exerts these biological actions via vitamin D receptors (VDRs), ligand-activated transcription factors that regulate the expression of hundreds of genes involved in metabolism. Consequently, alterations in vitamin D action, such as vitamin D deficiency, have been linked to numerous poor health outcomes in children, such as obesity, type 2 diabetes, hypertension, autoimmune disease, asthma, and multiple cancers [1,2,3,4,5,6,7,8,9].

The number of children developing obesity is increasing at an alarming rate, with up to one in five children in the U.S. affected [10]. More worrisome, obesity also increases a child’s risk of serious comorbidities, such as cardiovascular disease and type 2 diabetes [11]. Obesity is consistently associated with lower circulating concentrations of 25-hydroxyvitamin D (25OHD), the major circulating metabolite used clinically to assess vitamin D status [7]. Several mechanisms have been proposed to explain this association, including volumetric dilution, adipose tissue sequestration of vitamin D, altered hepatic and adipose vitamin D metabolism, and lifestyle differences [12]. However, the clinical consequences of this relationship remain incompletely understood due to the likelihood that circulating 25OHD represents a storage form of vitamin D rather than a true reflection of vitamin D action.

Currently, 25OHD levels are commonly used as a screening and treatment monitoring tool for vitamin D deficiency. While serum 25OHD correlates well with traditional physiological markers of vitamin D action in the lean population, such as serum calcium, phosphorus, parathyroid hormone, and overall bone health, this relationship does not parallel 25OHD levels in individuals with obesity [13,14,15,16,17]. This raises the possibility that circulating 25OHD concentrations may not accurately reflect vitamin D action in obesity. Because vitamin D action occurs through VDR-mediated transcriptional regulation within various tissues, evaluating expression of VDR-target genes may provide additional insight into tissue-specific vitamin D pathway biology.

There are many tissue-specific genes implicated in the regulation of the components of the vitamin D pathway that determine subsequent vitamin D action. To examine vitamin D activity across metabolically relevant tissues, we selected genes with established evidence of direct or indirect regulation by VDR and associations of vitamin D deficiency-related processes within each tissue. In intestinal tissue, we examined *TRPV6*, a classically associated transcriptional target of VDR involved in calcium homeostasis; *S100G*, also involved with vitamin D-mediated intracellular calcium transport; and *VDR* itself [18,19,20,21,22,23,24,25,26,27,28,29,30,31]. In adipose tissue, we evaluated *VDR*, the central mediator of vitamin D signaling; *TLR4*, an important regulator of innate immunity and inflammation modulated by vitamin D signaling; and *THBD*, a vitamin D-responsive gene involved in blood clotting [18,19,20,21,22,23,24,25,26,27,28,29,30,31]. Unlike the classic intestinal relationships of vitamin D and *TRPV6* and *S100G*, the relationships between *TLR4* and *THBD* and vitamin D are less direct and reflect broader biological interactions. Together, these genes are involved in complementary components of vitamin D-responsive metabolic pathways.

Although previous studies have examined VDR-target gene expression within isolated tissues, none have systematically evaluated the correlation of VDR-target gene expression level across multiple metabolically relevant human tissues in adolescents with severe obesity or examined its relationship with serum 25OHD levels. Here, we describe tissue-specific expression of VDR-target genes in visceral adipose tissue, subcutaneous adipose tissue, and small intestine obtained from adolescents undergoing bariatric surgery and compared these molecular measures with serum 25OHD concentrations. We hypothesized that VDR-pathway gene expression would correlate within metabolically relevant tissues but would not be significantly associated with circulating serum 25OHD levels. This would suggest that circulating vitamin D (25OHD) levels incompletely reflect vitamin D pathway biology in severe obesity.

## 2. Materials and Methods

### 2.1. Study Design and Participants

This study was a secondary analysis of banked biospecimens, including subcutaneous adipose tissue (SAT), visceral adipose tissue (VAT), tissue from the proximal end of the small intestine, and blood samples. The banked tissues and serum samples were obtained from adolescents aged 13–18 years with severe obesity undergoing elective bariatric surgery (Roux-en-Y gastric bypass) at Texas Children’s Hospital (Houston, TX, USA). Immediately prior to surgery, demographic and anthropometric data were collected, which included age, sex, race/ethnicity, and body mass index (BMI). All patients or their legal guardians provided informed consent as approved by the Institutional Review Board of Baylor College of Medicine. As this study was a secondary analysis of previously collected biologic samples under an earlier study, sample availability varied among tissues, resulting in differing sample sizes across experiments.

### 2.2. Tissue Collection and Serum 25-Hydroxyvitamin D (25OHD) Analysis

At the time of surgery, SAT, VAT, and proximal small intestinal tissues were obtained and immediately frozen in liquid nitrogen and stored at −80 °C until RNA extraction. In addition, 20 mL of venous blood was collected for analysis of serum 25OHD levels. Serum samples were analyzed by Texas Children’s Hospital pathology laboratory via chemiluminescent microparticle immunoassay according to the manufacturer’s standardized protocol.

### 2.3. RNA Extraction and Quantitative Real-Time PCR

Tissue RNA was extracted using a RNeasy Kit (Qiagen, Germantown, MD, USA). RNA concentration and purity were assessed using a NanoDrop spectrophotometer (Thermo Fisher Scientific, Waltham, MA, USA). cDNA was synthesized from RNA, and real-time quantitative PCR (qPCR) was performed using a TaqMan 7900 sequence detection system with TaqMan universal PCR master mix and TaqMan gene expression assays (Applied Biosystems, Foster City, CA, USA). Reactions were performed in triplicate. A Ct cutoff of 30 was used for gene-expression analyses; samples with Ct values > 30 or undetectable expression were excluded from the corresponding gene-specific analysis. Technical replicates demonstrating discordant amplification were repeated. Target gene Ct values were normalized to the mean Ct of the two tissue-specific housekeeping genes listed in Table 1 to obtain ΔCt values (*GAPDH* and *RPLP0* for adipose and *GAPDH* and *ACTB* for intestinal tissue). Relative gene-expression values were subsequently calculated using the comparative Ct (2^−ΔΔCt^) method as described by Livak et al. [32]. Relative expression values derived from the ΔΔCT method were used for subsequent analyses. For graphical presentation and statistical analyses, expression values were then multiplied by 100. Expression values are therefore reported in arbitrary units (a.u.).

### 2.4. Initial Gene Selection and Exploratory Analyses of Additional Candidate Genes

We selected the genes for analysis based on existing studies that had previously shown either (1) VDR binding to the promoter of the gene, (2) changes in gene expression levels in differing vitamin D environments, or (3) an association with vitamin D deficiency-related processes, such as *TRPV6* and *S100G* in intestinal calcium transport and *TLR4* and *THBD* in vitamin D-associated inflammatory and vascular pathways (Table 1) [18,19,20,21,22,23,24,25,26,27,28,29,30,31]. Since gene regulation is tissue-specific, we analyzed the expression of *VDR* in several vitamin D pathway-related tissues.

Exploratory analyses were subsequently performed to expand the analysis to include additional VDR pathway genes despite their previously limited studies, such as *CEBPA* and *PPARG* in the adipose tissues and *CYP24A1* in the intestinal tissue. As these were exploratory, post hoc analyses, they were not part of the original analytic plan.

### 2.5. Statistical Analysis

Continuous variables are presented as the mean ± standard deviation (SD). Categorical variables are presented as frequencies and percentages. The data for qPCR measurements were analyzed utilizing simple linear regression analysis, scatter plot matrices, and heat plots using GraphPad Prism (version 11.1.0, Boston MA USA) and R software (version 4.4.2, Vienna, Austria). Regression results were reported using coefficients of determination (R^2^), regression slopes with 95% confidence intervals, and *p*-values. *p*-values < 0.05 were considered to be statistically significant. For analyses of tissue-specific gene expression, relative expression values calculated using the ΔΔCT method were used. Samples with Ct values > 30 or undetectable target gene expression were excluded from the corresponding gene-specific analysis. Formal testing of normality was not performed. Adjustment for multiple comparisons was also not performed given the exploratory nature of the study and that the candidate gene selection was based upon established or proposed biological relationships with vitamin D activity in the literature. Given the exploratory and hypothesis-generating nature of the analyses, reported *p*-values should be interpreted cautiously, particularly for the post hoc exploratory analyses, and statistically significant findings require validation in independent cohorts.

Given the nature of the study as a secondary analysis of archived biospecimens, sample availability and quality varied by tissue. Some banked specimens were degraded or otherwise unsuitable for RNA extraction and gene expression analysis, while in others, expression of individual genes was not detected. In addition, only biological samples with measurements for both variables were included in the correlation analyses, resulting in variation in sample size across genes and tissues.

For exploratory cross-tissue analyses, participant-level mean ΔCt values were calculated within each tissue. The VAT and SAT means each included the available ΔCt values for *PPARG*, *CEBPA*, *THBD*, *TLR4*, and *VDR*, whereas the intestinal mean included the values for *S100G*, *CYP24A1*, *TRPV6*, and *VDR.* Means were calculated using available gene-expression measurements for each participant, with missing gene measurements omitted from the calculation. These exploratory analyses were generated using R statistical software from the available relative gene-expression data. Specifically, Pearson correlations among tissue-specific mean ΔCt values were calculated using pairwise complete observations. Exploratory correlations among individual tissue-specific gene-expression measures were similarly evaluated using Pearson correlations of ΔCt values with pairwise complete observations. The individual gene correlation matrix was visualized using the corrplot package, with hierarchical clustering used to order the displayed variables.

## 3. Results

### 3.1. Study Cohort

Samples were obtained from 154 adolescents undergoing elective Roux-en-Y gastric bypass surgery at a large, tertiary children’s hospital. Patient demographic and clinical characteristics are summarized in Table 2. Vitamin D supplementation status was not available for this secondary analysis. Our patient population mirrors national trends in pediatric metabolic and bariatric surgery demographic data with a mean (SD) age of 17.1 (1.2) years, a predominantly female population (77%), and average BMI greater than 50 kg/m^2^ [33]. Contrary to national trends, our patients included a racially and ethnically diverse group of adolescents, with the majority identifying as Hispanic (34%) and African American or Black (33%).

### 3.2. Circulating Serum 25OHD Levels Are Inversely Associated with BMI in Obesity

**Figure 1:**
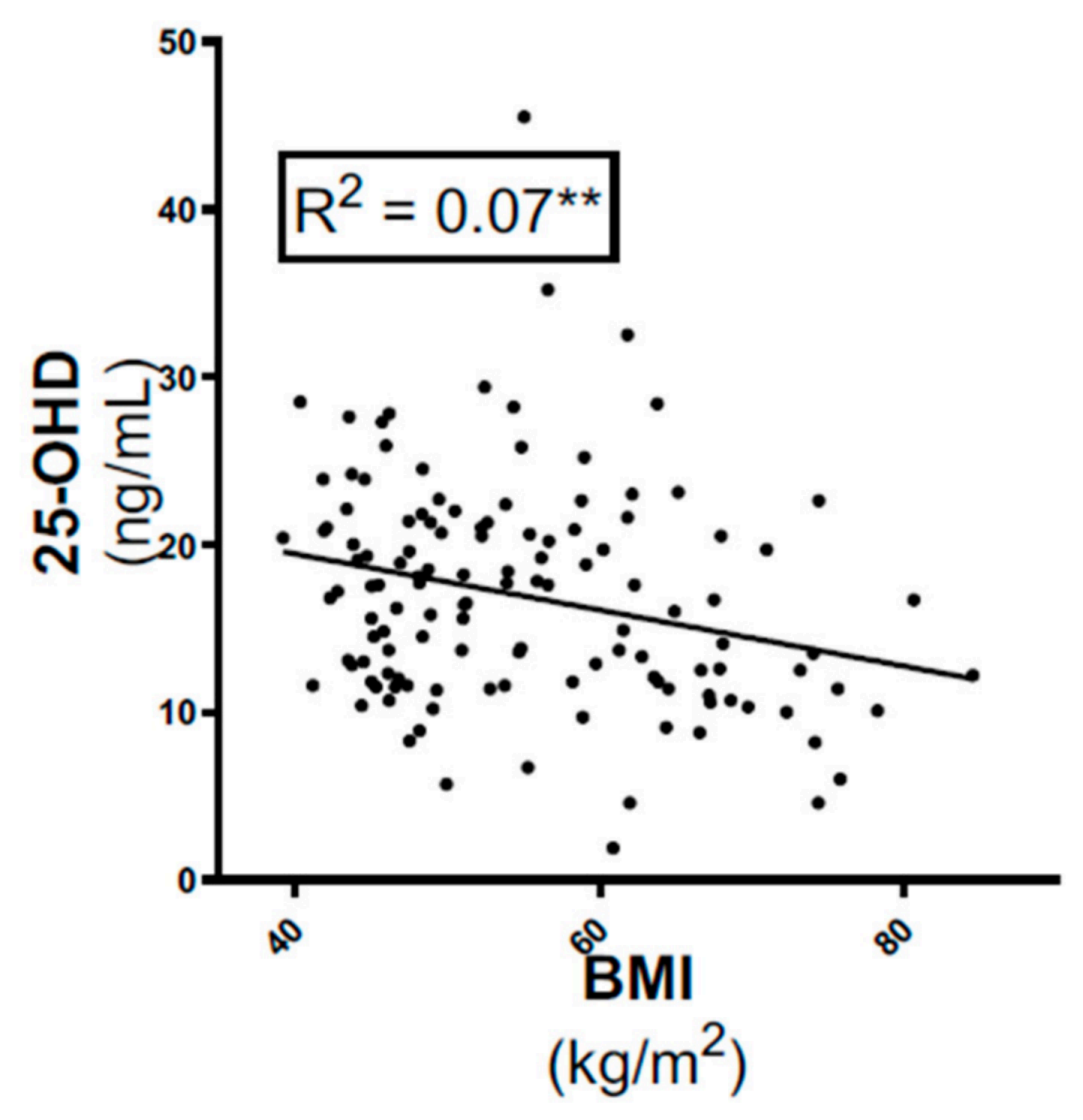
Simple linear regression of circulating serum vitamin 25OHD levels vs. BMI of adolescents undergoing bariatric surgery. N = 154. *R*^2^ = 0.07, slope −0.17, 95% CI −0.28–−0.06, *p* = 0.004, *p* < 0.01 **.

There was a significant inverse correlation between serum 25OHD levels and BMI (Figure 1). The mean (SD) 25OHD level was 16.9 (6.7) ng/mL, with 61% of patients having a vitamin D deficiency or a 25OHD level lower than 20 ng/mL (Table 2), consistent with previous studies demonstrating low serum 25OHD levels or a higher prevalence of vitamin D deficiency among individuals with obesity.

### 3.3. Vitamin D Receptor (VDR)-Target Genes Demonstrate Correlated Expression Patterns Within Tissues

To determine vitamin D action in the body on a molecular level, expression patterns of selected VDR-target genes were evaluated across various tissue types. We selected the genes for analysis based on the existing literature, as described above and depicted in Table 1 [19,20,21,22,23,24,25,26,27,28,29,30,31,32]. Since gene regulation is tissue-specific, we analyzed the expression of VDR in several vitamin D pathway-related tissues, specifically subcutaneous adipose tissue (SAT), visceral adipose tissue (VAT), and intestinal tissue. Tissue-specific gene expressions were compared to VDR expression within each tissue to appreciate the changes in expression levels of these tissue-specific genes in relation to genomic actions of VDR. Sample sizes varied among analyses due to differences in archived tissue availability and quality leading to exclusion of samples without expression measurements. In addition, only samples with measurements for both variables were included for correlation analyses. Individual sample sizes are reported in the corresponding figure legends.

**Figure 2:**
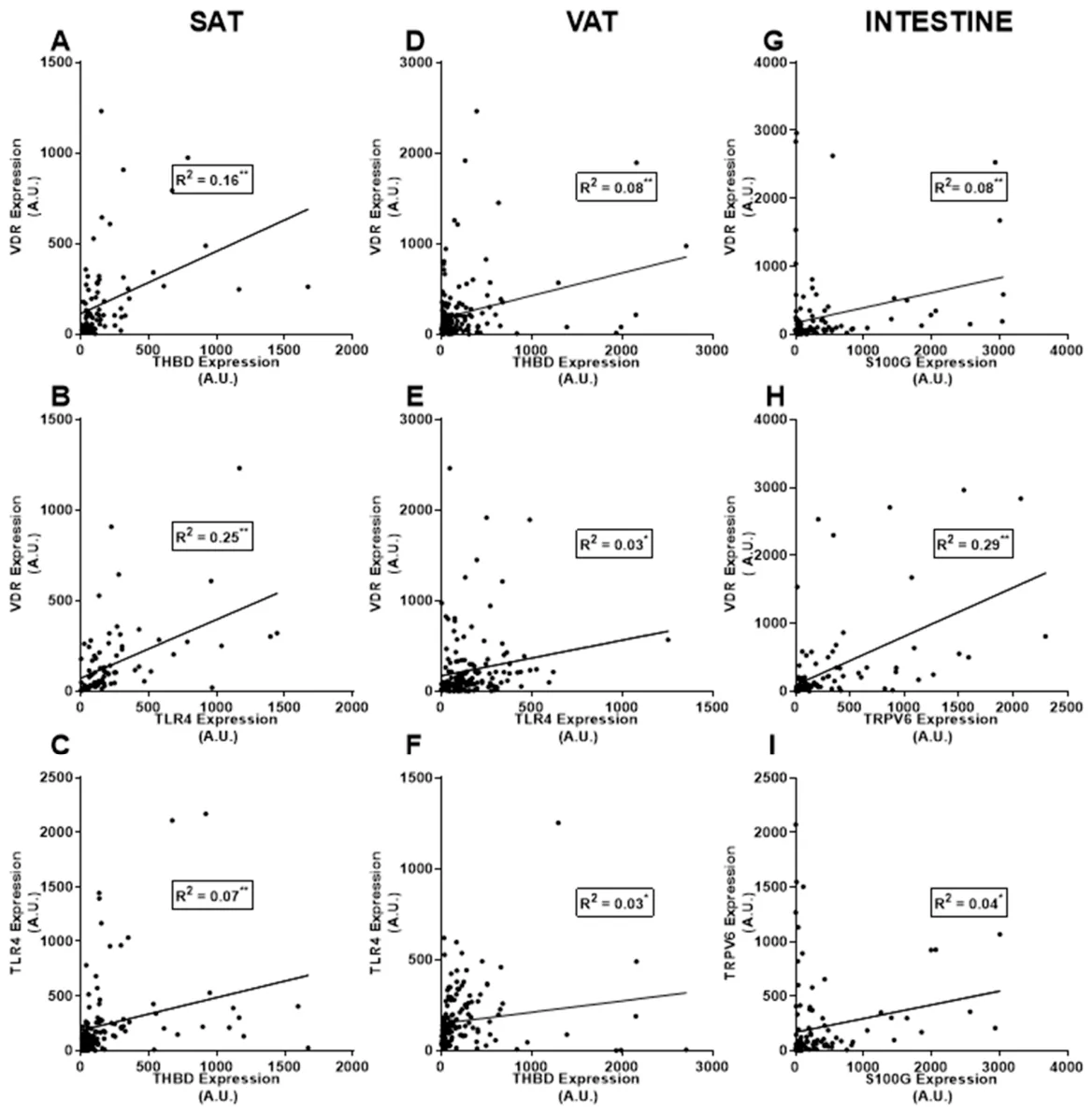
VDR-target gene expression simple linear regression analyses. (**A**–**C**) SAT correlations of *VDR* versus *THBD* (*n* = 88, R^2^ = 0.16, slope 0.35, 95% CI 0.17–0.52, *p =* 0.0001) and *TLR4* gene expression (*n* = 87, *R*^2^ = 0.25, slope 0.33, 95% CI 0.20–0.45, *p* < 0.0001) and *THBD* versus *TLR4* gene expression (*n* = 121, *R*^2^ = 0.07, slope 0.30, 95% CI 0.10–0.50, *p =* 0.004). (**D**–**F**) VAT correlations of *VDR* versus *THBD* (*n* = 154, *R*^2^ = 0.08, slope 0.25, 95% CI 0.12–0.38, *p =* 0.0003) and *TLR4* gene expression (*n* = 154, *R*^2^ = 0.03, slope 0.4, 95% CI 0.03–0.76, *p =* 0.04) and *THBD* versus *TLR4* gene expression (*n* = 154, *R*^2^ = 0.03, slope 0.06, 95% CI 0.004–0.12, *p =* 0.04) (**G**–**I**) Intestinal correlations of *VDR* versus *S100G* (*n* = 116, *R*^2^ = 0.08, slope 0.22, 95% CI 0.08–0.36, *p =* 0.003) and *TRPV6* gene expression (*n* = 116, *R*^2^ = 0.29, slope 0.72, 95% CI 0.51–0.92, *p* < 0.0001) and *S100G* versus *TRPV6* gene expression (*n* = 107, *R*^2^ = 0.04, slope 0.13, 95% CI 0.01–0.25, *p* = 0.03). Gene-expression values are reported in arbitrary units and represent relative expression calculated using the comparative Ct (^2^−ΔΔCt^) method and multiplied by 100 with target gene Ct values normalized to the mean Ct of the two tissue-specific housekeeping genes listed in Table 1. * *p* < 0.05. ** *p* < 0.01.

Within both adipose tissues, expression of VDR pathway genes demonstrated correlated expression patterns. *VDR* expression was significantly correlated with both *THBD* and *TLR4*, while *THBD* and *TLR4* expression were also significantly associated with one another (Figure 2). A similar pattern was observed within intestinal tissue. Expression of *VDR* was significantly correlated with both *TRPV6* and *S100G*, and *TRPV6* expression was significantly correlated with *S100G* expression (Figure 2). These correlated expression patterns were observed despite the genes representing distinct biological pathways involved in calcium transport, immune regulation, and vascular homeostasis.

### 3.4. Exploratory Analyses of Additional Vitamin D-Responsive Genes

**Figure 3:**
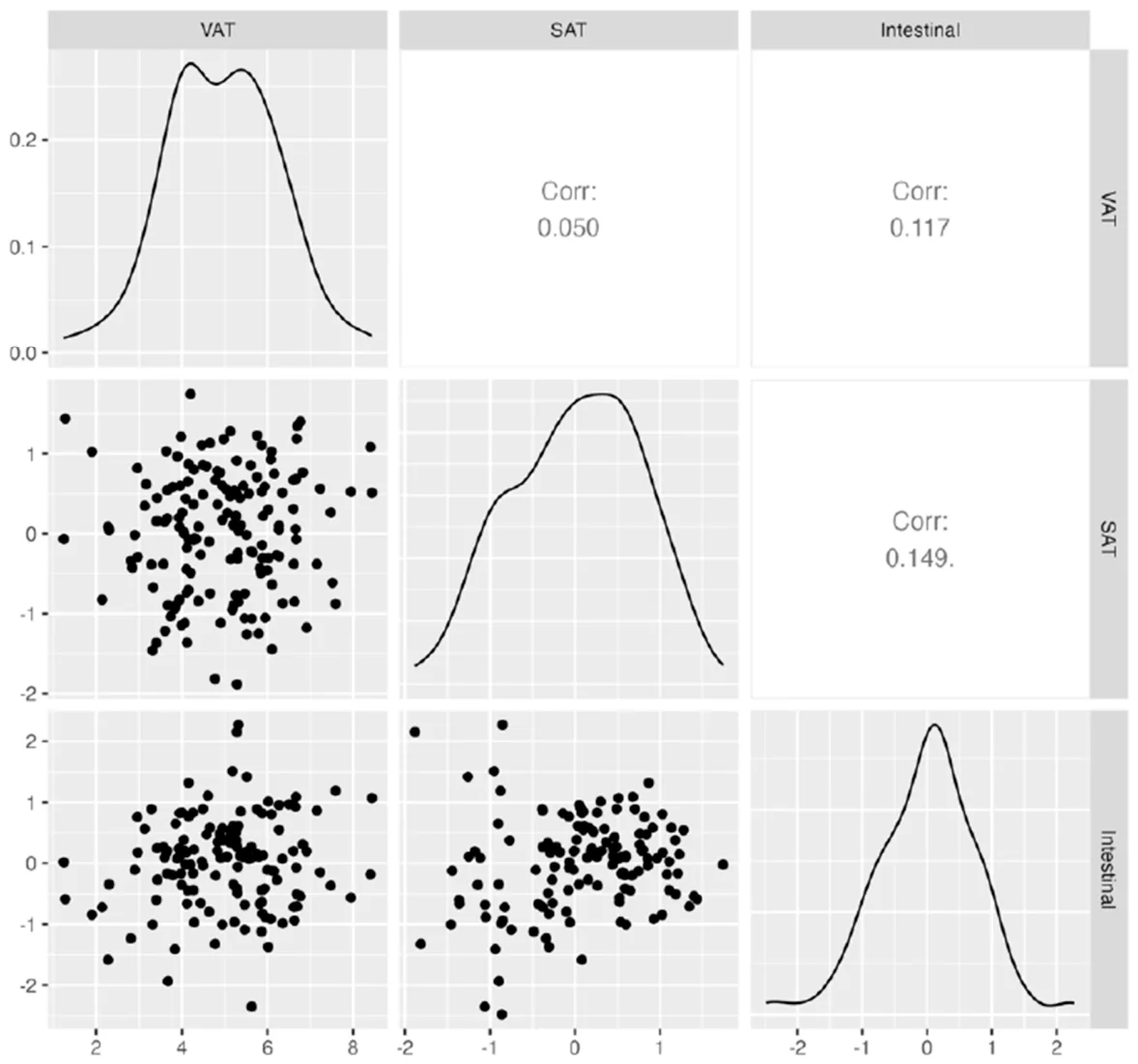
Pairwise relationships among mean VDR target gene-expression measures across metabolically relevant tissues. Using R statistical software, a tissue-specific mean ΔCt value for each participant was calculated from the available gene-expression measurements within each tissue with missing values excluded. Relative gene expression was derived from qPCR measurements normalized to the tissue-specific housekeeping genes as described in the Methods. Samples with Ct values > 30 or undetectable target gene expression were excluded from the corresponding analysis. The VAT mean included *PPARG*, *TLR4*, *THBD*, *VDR*, and *CEBPA*; the SAT mean included *PPARG*, *CEBPA*, *THBD*, *VDR*, and *TLR4*; and the intestinal mean included *S100G*, *CYP24A1*, *VDR*, and *TRPV6.* Pearson correlations were calculated between tissue-specific mean ΔCt values using pairwise complete observations. VAT versus SAT: *r* = 0.05, *n* = 154, *p* = 0.5363; VAT versus intestine: *r* = 0.12, *n* = 136, *p* = 0.1736; and SAT versus intestine: *r* = 0.15, *n* = 132, *p* = 0.0873. Diagonal panels display the distribution of each tissue-specific mean, lower panels display pairwise scatterplots, and upper panels display Pearson correlation coefficients. VAT, visceral adipose tissue; SAT, subcutaneous adipose tissue.

**Figure 4:**
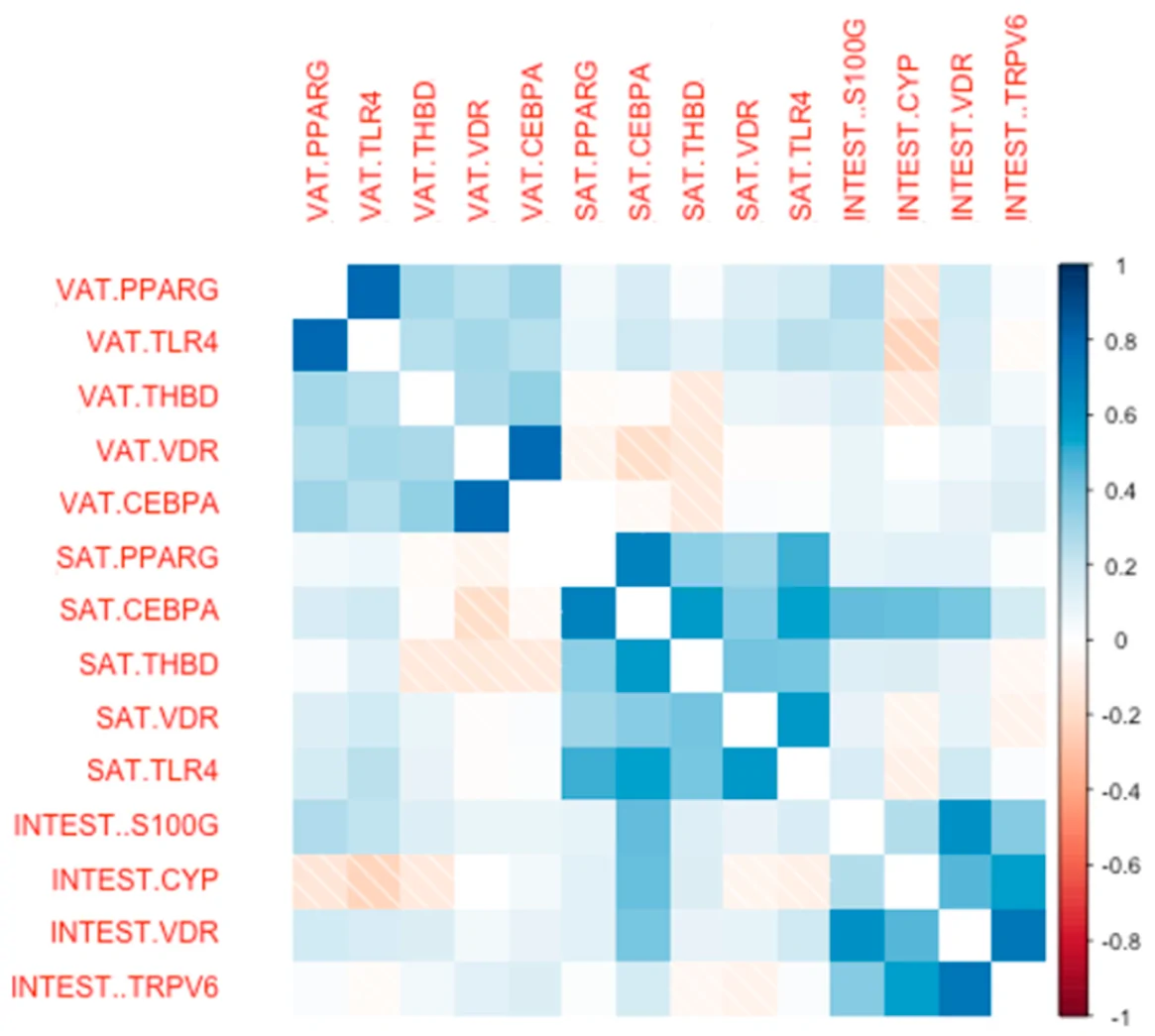
Correlation heatmap of VDR-target gene expression across metabolically relevant tissues. Pearson correlation coefficients were calculated in R statistical software from ΔCt gene-expression values for the indicated genes in visceral adipose tissue (VAT), subcutaneous adipose tissue (SAT), and intestinal tissue as described in the Methods. Samples with Ct values > 30 or undetectable target gene expression were excluded from the corresponding gene-specific analysis. Missing observations were handled using pairwise complete observations, such that each correlation included participants with available expression measurements for both variables in the corresponding comparison. Therefore, sample sizes varied across pairwise correlations. No additional transformation or standardization was applied within this correlation analysis. The resulting correlation matrix was visualized using the corrplot package, with hierarchical clustering used to order the displayed variables. Correlation coefficients range from −1 to +1, with blue indicating positive correlations, red indicating negative correlations, and values approaching white indicating weaker correlations.

**Figure A1:**
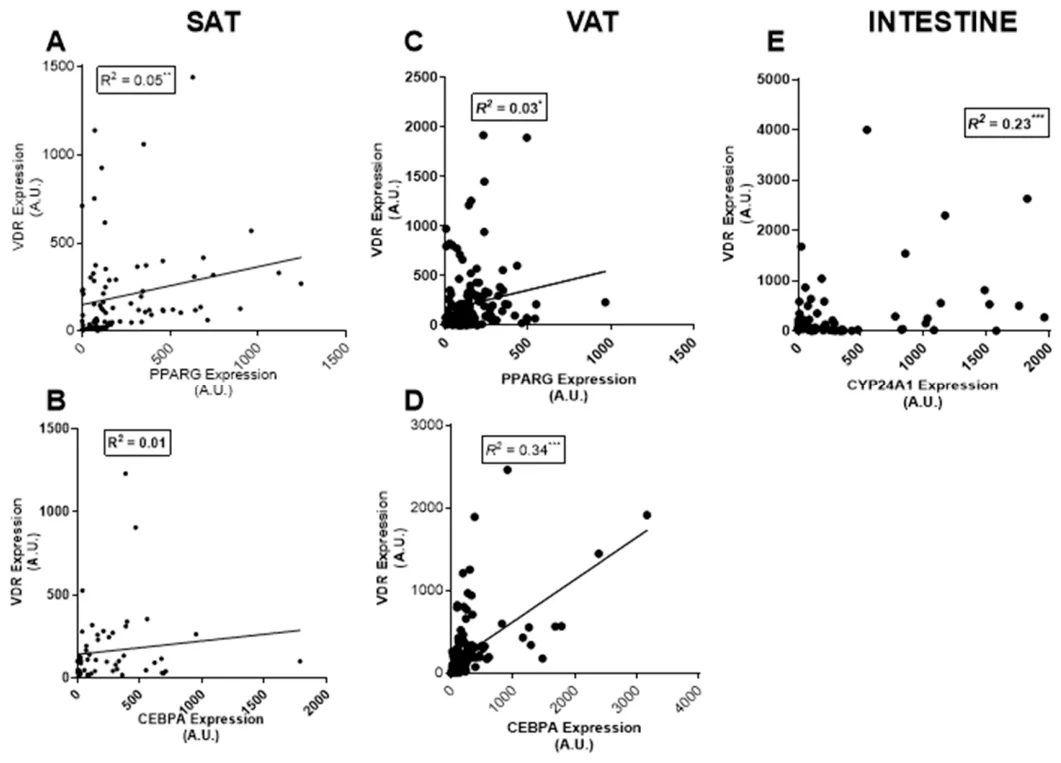
Additional VDR-target gene expression correlations. (**A**,**B**) SAT correlations of *VDR* versus *PPARG* and *CEBPA* gene expression. (**C**,**D**) VAT correlations of *VDR* versus *PPARG* and *CEBPA* gene expression. (**E**) Intestinal correlations of *VDR* versus *CYP24A1* gene expression. Gene-expression values are reported in arbitrary units and represent relative expression calculated using the comparative Ct (^2^−ΔΔCt^) method and multiplied by 100 with target gene Ct values normalized to the mean Ct of the two tissue-specific housekeeping genes listed in Table 1. * *p* < 0.05. ** *p* < 0.01. *** *p* < 0.001. *n* = 75–121 per gene.

Given these strong findings from the primary analyses, additional exploratory analyses were performed to include additional VDR pathway genes. Within both adipose tissues, *VDR* expression significantly correlated with *PPARG*, whereas significant associations between *VDR* and *CEBPA* were observed only in VAT (Appendix A Figure A1). In intestinal tissue, *VDR* expression also demonstrated a significant positive association with *CYP24A1*, a well-established vitamin D-responsive gene (Appendix A Figure A1). Exploratory comparisons of tissue-specific gene expression demonstrated no statistically significant correlations between mean ΔCt values across tissues (VAT–SAT: *r* = 0.05, *p* = 0.5363; VAT–intestine: *r* = 0.12, *p* = 0.1736; SAT–intestine: *r* = 0.15, *p* = 0.0873) (Figure 3 and Figure 4).

### 3.5. Tissue-Specific VDR Pathway Gene Expression Is Not Significantly Associated with Serum 25OHD Levels

**Figure 5:**
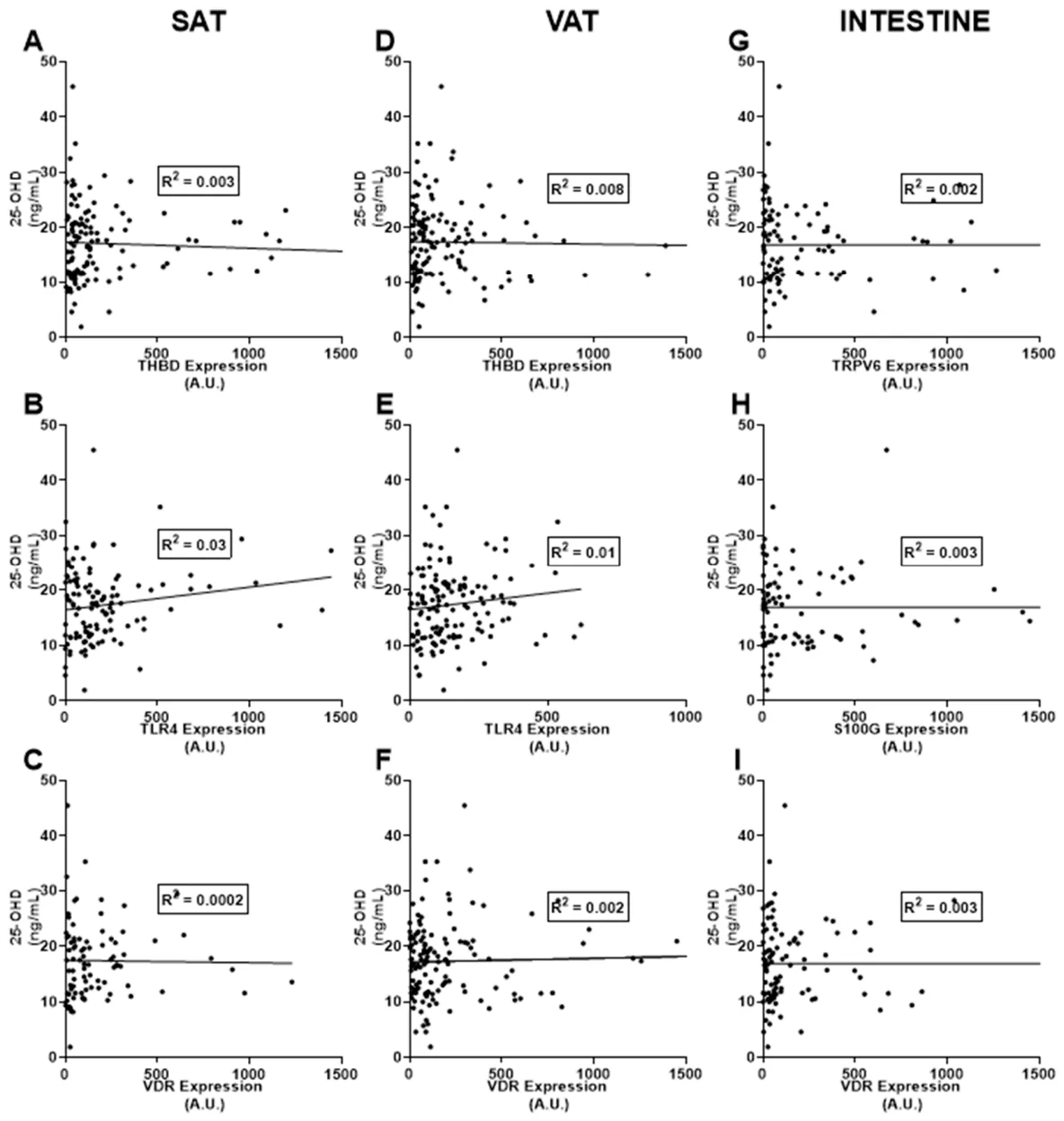
VDR-target gene expression correlations compared to serum 25OHD levels. In SAT (**A**–**C**), 25OHD level versus *THBD* (*n* = 121, *R*^2^ = 0.003, slope −0.001, 95% CI −0.005–0.003, *p* = 0.56), *TLR4* (*n* = 117, *R*^2^ = 0.03, slope 0.004, 95% CI −0.0005–0.009, *p* = 0.08), and *VDR* (*n* = 87, *R*^2^ = 0.0002, slope −0.0004, 95% CI −0.007–0.006, *p* = 0.90). In VAT (**D**–**F**), 25OHD level versus *THBD* (*n* = 140, *R*^2^ = 0.0008, slope −0.0005, 95% CI −0.003–0.002, *p* = 0.74), *TLR4* (*n* = 140, *R*^2^ = 0.01, slope 0.006, 95% CI −0.003–0.015, *p* = 0.19), and *VDR* (*n* = 138, *R*^2^ = 0.002, slope 0.0007, 95% CI −0.002–0.004, *p* = 0.65). In intestinal tissue (**G**–**I**), 25OHD level versus *TRPV6* (*n* = 113, *R*^2^ = 0.002, slope 5.64 × 10^−6^, 95% CI −1.36 × 10^−5^–8.72 × 10^−6^, *p* = 0.67), *S100G* (*n* = 113, *R*^2^ = 0.003, slope −2.19 × 10^−7^, 95% CI −1.02 × 10^−6^–5.83 × 10^−7^, *p* = 0.59), and *VDR* (*n* = 113, *R*^2^ = 0.003, slope −7.3 × 10^−7^, 95% CI −3.42 × 10^−6^–1.97 × 10^−6^, *p* = 0.59). Gene-expression values are reported in arbitrary units and represent relative expression calculated using the comparative Ct (^2^−ΔΔCt^) method and multiplied by 100 with target gene Ct values normalized to the mean Ct of the two tissue-specific housekeeping genes listed in Table 1.

To determine whether circulating vitamin D concentrations reflected vitamin D action at the tissue level, expression of each tissue-specific VDR pathway gene was compared with circulating serum 25OHD levels. No statistically significant associations were identified between serum 25OHD concentrations and expression of VDR-target genes in SAT, VAT, or intestinal tissue (Figure 5). This lack of association was observed across genes representing multiple biological pathways and tissue types.

## 4. Discussion

In this secondary analysis of adolescents with severe obesity undergoing bariatric surgery, we evaluated expression of tissue-specific vitamin D receptor (VDR) pathway genes across multiple metabolically relevant tissues and examined their relationship with circulating serum 25-hydroxyvitamin D (25OHD) concentrations. To our knowledge, our studies are the first to systematically and simultaneously investigate correlated VDR-target gene expression across subcutaneous adipose tissue (SAT), visceral adipose tissue (VAT), and intestinal tissue obtained from adolescents with severe obesity. We found that these VDR-target genes demonstrated correlated expression patterns across these multiple tissues. In contrast, our findings did not show that VDR-target gene expression was significantly associated with circulating serum 25OHD levels, coinciding with increasing evidence regarding the inconsistency of utilizing circulating serum 25OHD as a marker of vitamin D status in obesity [14,15,16,17].

Our findings extend previous work that had examined vitamin D signaling and VDR-target gene expression within isolated tissues. Specifically, previous human studies have shown relationships between vitamin D status and adipose tissue VDR or vitamin D-responsive gene expression. Clemente-Postigo et al. demonstrated that circulating vitamin D status and adipose VDR expression may vary in individuals with obesity and type 2 diabetes [23]. Carlberg et al. also demonstrated varied changes in circulating vitamin D levels versus vitamin D target gene expression in response to vitamin D supplementation [25]. However, Ryynänen et al. revealed that vitamin D target gene expression in adipose tissue may respond to vitamin D supplementation [24]. Together, these studies indicate that the current clinical marker of vitamin D status may not fully reflect vitamin D action in individuals with obesity.

Interestingly, the correlated expression patterns in our study occurred within tissues of differing metabolic functions—two which store vitamin D (subcutaneous and visceral adipose tissue) and one which does not (intestinal)—suggesting conserved tissue-specific VDR pathways. VDR is expressed in both adipose and intestinal tissues with vitamin D signaling occurring in distinct biological processes [18,19,20,21,22,23,24,25,26,27,28,29,30,31]. In intestinal tissue, VDR-responsive genes, such as *TRPV6* and *S100G/calbindin-D9k*, are involved in calcium transport and are well-established within VDR signaling [19,20]. In adipose tissue, vitamin D signaling is more complex and has been associated with adipogenesis, lipid metabolism, inflammatory signaling, and local vitamin D metabolism [21,22,28,29,30,31]. The VDR pathway genes evaluated within the tissues included known vitamin D-responsive genes as well as genes with more indirect relationships to VDR signaling. For example, *TRPV6* and *S100G* have established roles in vitamin D-regulated calcium homeostasis [19,20], whereas *TLR4* is linked to immunoregulatory effects of vitamin D [26,27]. However, the variable strength of the observed associations further supports describing these findings as correlated expression patterns rather than coordinated regulation. The exploratory analyses of *CEBPA*, *PPARG*, and *CYP24A1* should be considered hypothesis-generating and require independent validation.

Several mechanisms may contribute to the lack of a statistically significant association between circulating 25OHD concentrations and tissue-specific VDR-target gene expression levels, such as altered vitamin D bioavailability through volumetric dilution, sequestration of this fat-soluble vitamin within adipose tissue, obesity-related inflammation, and possible epigenetic regulation of VDR-responsive genes [33,34,35,36]. Together, these mechanisms provide biologically plausible explanations for why circulating 25OHD concentrations may not correlate with tissue-specific VDR-target gene expression in obesity. Genetic variation, including VDR polymorphisms and allele-specific transcriptional regulation, represents an additional potential contributor that was not assessed in the present study. Importantly, these mechanisms were not directly examined in the present study and warrant further investigation.

From a clinical perspective, serum 25OHD remains the established biomarker to assess vitamin D status, but the absence of statistically significant associations between serum 25OHD and VDR-target gene expression across multiple tissues raises the possibility that circulating vitamin D may not fully reflect VDR pathway biology in individuals with severe obesity.

This study has many strengths, including the use of multiple metabolically relevant tissues within the same cohort and that the samples were human biospecimens from adolescents with severe obesity. In addition, our patient population uniquely had higher rates of bariatric surgery among minority groups despite previously reported racial and ethnic disparities between rates of pediatric obesity and access to bariatric surgery [37]. However, this study population also represents a highly selected population of adolescents with severe obesity already approved for Roux-en-Y gastric bypass surgery at a single tertiary-care center. The cohort was predominantly female and had a mean BMI > 50 kg/m^2^. Accordingly, these findings cannot be extrapolated to pediatric patients with milder obesity or non-surgical pediatric populations without additional validation.

Several additional limitations should be considered given the nature of a secondary analysis of banked biospecimens from a previous protocol. Sample sizes limited opportunities to run additional analyses or interrogate additional clinical variables. Specifically, sample sizes were limited and varied across tissue- and gene-specific analyses because of tissue quality and reliable detection of individual gene expression. Furthermore, additional demographic information or the ability to obtain post-surgical data as an added cohort for direct comparison was unavailable given the nature of a secondary analysis. Vitamin D supplementation and other potential determinants of vitamin D status, such as seasonality, dietary vitamin D intake, renal and hepatic function, inflammatory status, medication usage, and sun exposure, were also unavailable for this secondary analysis. The limited availability of the original participant dataset also did not allow for multivariable adjustment for other potential confounders that may influence serum 25OHD levels, such as age, sex, BMI, and race/ethnicity.

Additionally, the statistical analyses did not include formal testing of normality or adjustment for multiple comparisons. Due to the nature of a secondary analysis and insufficient access to the original individual-level dataset, an alternative correlation analysis or Spearman sensitivity analysis could not be performed. Although the candidate genes were selected based upon plausibility from established literature and the analyses were exploratory, the possibility of type 1 errors cannot be excluded, which is particularly important for post hoc exploratory analyses. Thus, these findings should be regarded as hypothesis-generating until validated in independent cohorts. Furthermore, the absence of statistical significance should not be interpreted as evidence that no relationship exists, particularly for analyses with varying sample sizes. Effect estimates and their 95% confidence intervals should therefore be considered alongside *p*-values when interpreting these associations.

Finally, the cross-sectional design precludes determination of temporal or causal relationships between serum 25OHD and VDR-target gene expression. Relative gene expression represents only one component of vitamin D signaling and does not fully reflect activity. Protein abundance, VDR activity, downstream physiological responses, circulating 1,25-dihydroxyvitamin D (1,25OHD), and parathyroid hormone (PTH) were not assessed. Consequently, the transcriptional measures evaluated in this study should not be interpreted as direct measures of tissue-level vitamin D action. Finally, the ΔΔCt approach quantified total relative transcript abundance and did not assess allele-specific expression; thus, potential contributions of genetic variation to individual differences in VDR-target gene expression could not be evaluated. Future studies integrating genotyping with allele-specific transcriptional analyses may provide additional insight into genetic determinants of tissue-specific vitamin D activity.

Prospective studies are needed to investigate the effect of vitamin D supplementation or treatment in a dose-dependent manner on VDR-target gene expression levels. Ideally, these studies would evaluate the same VDR pathway genes before and after standardized vitamin D supplementation while concurrently measuring serum 25OHD, 1,25OHD, PTH, inflammatory and metabolic markers, and relevant clinical variables. In addition, pediatric patients across a broader range of obesity severity and appropriate comparison groups would help determine the generalizability of this study.

## 5. Conclusions

In adolescents with severe obesity, VDR pathway genes demonstrated correlated tissue-specific expression across adipose and intestinal tissues but were not associated with circulating serum 25-hydroxyvitamin D concentrations. These findings highlight the complexity of relating serum vitamin D status to tissue-level molecular pathways and suggest that circulating serum 25OHD may not fully reflect vitamin D biology in severe obesity. This supports further investigation into how vitamin D signaling is regulated in obesity. Prospective studies incorporating broader clinical phenotyping and longitudinal measures of transcriptional, protein, hormonal, and functional responses before and after vitamin D supplementation are needed to improve our understanding of vitamin D biology and therapeutic responses in obesity.

## Figures and Tables

**Table 1:** Genes chosen for analysis.

	Adipose Tissues	Intestinal Tissues
**Housekeeping Genes**		
***GAPDH***	**×**	**×**
***RPLPO***	**×**	
***ACTB***		**×**
**VDR-Target Genes**		
***VDR***	**×**	**×**
***TLR4***	**×**	
***THBD***	**×**	
***TRPV6***		**×**
***S100G***		**×**

**Table 2:** Descriptive characteristics of overall study cohort of adolescent patients. N = 154. Data are presented as mean or number (*n*) with respective (SD) or (%), as indicated. Sample sizes for individual tissue and gene-expression analyses varied based on quality and availability of archived specimens from each participant and are reported with corresponding analyses.

Characteristics	*n* (%) or Mean (SD)
**Age (years)**	**17.12 (1.18)**
**Gender**	
**Male**	**36 (23)**
**Female**	**118 (77)**
**BMI**	**55.07 (10.79)**
**Race or Ethnicity**	
**Hispanic**	**52 (34)**
**Black or African American**	**50 (33)**
**Non-Hispanic White**	**46 (30)**
**Asian**	**1 (0.6)**
**American Native or Pacific Islander**	**1 (0.6)**
**Unknown/Did Not Disclose**	**4 (2.6)**
**Vitamin 25OHD (ng/mL)**	
**Mean**	**16.93 (6.67)**
**Vitamin D deficient (25OHD < 20)**	**94 (61)**

## Data Availability

All of the deidentified individual participant data, molecular data, and statistical plans will be made immediately and indefinitely available following publication to any investigator whose proposed use of the data has been approved by an independent review committee identified for this purpose.

## Abbreviations

The following abbreviations are used in this manuscript:

VDR	Vitamin D receptor
25OHD	25-hydroxyvitamin D
1,25OHD	1,25-dihydroxyvitamin D
VAT	Visceral adipose tissue
SAT	Subcutaneous adipose tissue
BMI	Body mass index
qPCR	Quantitative PCR
SD	Standard deviation
